# Integrin-Targeting Strategies for Adenovirus Gene Therapy

**DOI:** 10.3390/v16050770

**Published:** 2024-05-13

**Authors:** Glen R. Nemerow

**Affiliations:** Department of Immunology, The Scripps Research Institute, 10666 North Torrey Pines Rd, La Jolla, CA 92037, USA; gnemerow@scripps.edu

**Keywords:** adenovirus, penton base, fiber, integrins, extracellular matrix proteins, nanoparticles, bioimaging

## Abstract

Numerous human adenovirus (AdV) types are endowed with arginine–glycine–aspartic acid (RGD) sequences that enable them to recognize vitronectin-binding (αv) integrins. These RGD-binding cell receptors mediate AdV entry into host cells, a crucial early step in virus infection. Integrin interactions with adenoviruses not only initiate receptor-mediated endocytosis but also facilitate AdV capsid disassembly, a prerequisite for membrane penetration by AdV protein VI. This review discusses fundamental aspects of AdV–host interactions mediated by integrins. Recent efforts to re-engineer AdV vectors and non-viral nanoparticles to target αv integrins for bioimaging and the eradication of cancer cells will also be discussed.

## 1. Introduction

**Figure 1:**
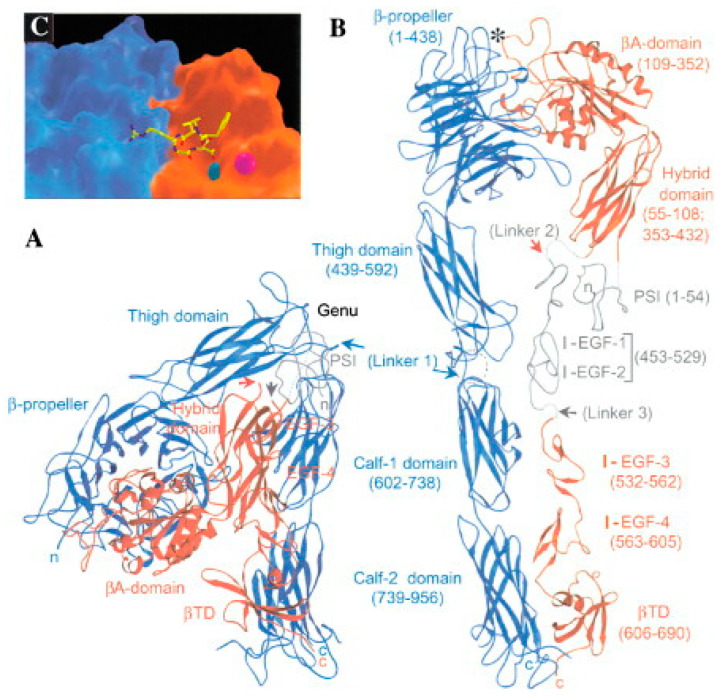
**Three-dimensional structure of integrin αvβ3.** The inactive (bent) form of the integrin (without its bound RGD ligand) is shown as a ribbon diagram with the αv subunit in blue and the β3 subunit in red (**A**). The C-terminus of these subunits are inserted into the cell membrane, whereas the N-terminal domains are involved in ligand binding. The extended (active) form of the integrin is shown in (**B**) with the location of the bound RGD ligand indicated by an *. A surface rendering of the alpha subunit (red) and beta subunit (blue) with the bound cyclo-RGDF peptide (stick figure) in the interface between the αv and β3 subunits is shown in (**C**). Reprinted by permission from Hynes, 2002, Cell press [3].

**Figure 2:**
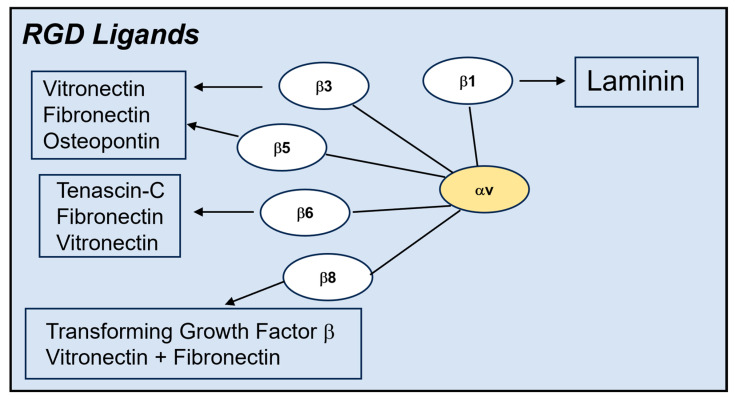
**Diagram of the αv integrin family and natural ligands.** αv integrins comprise five different heterodimeric receptors within the larger family of 24 known integrin molecules [3]. The majority of αv integrins recognize extracellular (ECM) ligands such as vitronectin and fibronectin that contain an RGD sequence. These receptor–ligand interactions foster cell adhesion and cell migration, processes crucial for maintaining normal cell homeostasis.

Long before the clever Ulysses used a hollow wooden horse to transport the Greek army into the city of Troy (epic poem by Virgil in the *Aeneid*), mammalian viruses adopted a similar strategy to invade host cells, albeit at the molecular level. Foremost among these molecular con artists are integrins, which are the focus of this review. Integrins are heterodimeric membrane glycoproteins consisting of an alpha and beta subunit that recognize a variety of extracellular matrix proteins (ECMs) [1] (Figure 1 and Figure 2). Ligand binding to these receptors involves the recognition of discreet amino acid sequences displayed on the surface of ECMs. For example, certain integrins latch onto the arginine, glycine, and aspartic acid (RGD) motifs displayed on vitronectin and fibronectin [2,3]. The binding of these ligands generally requires the presence of divalent metal cations such as magnesium or manganese. Initial ligand binding promotes transition of the integrin from an inactive (bent) to an active (extended) conformation [4]. Integrin ligation by ECMs can trigger cell spreading, cell adhesion, cell migration, and endocytosis [2,5]. Engagement of the beta integrin cytoplasmic domain with elements of the cell cytoskeleton facilitates these cellular processes [3,6].

In the early 1990s, new information, specifically in the realm of microbial pathogenesis, emerged concerning the role of integrins to mediate cell infection. Bacteria such as Yersinia [7] and Bordetella pertussis [8] were shown to bind to β1 and β2 integrins, respectively, and that these interactions led to pathogen uptake into susceptible host cells. Integrins were also reported to mediate cell binding of several mammalian viruses including echovirus 1 [9] and foot-and-mouth disease virus [10].

Knowledge of integrin association with diverse microbial pathogens served as an impetus for investigating whether human adenovirus also interacts with integrins. In this regard, very early reports indicated that human species C adenoviruses produced a so-called toxic factor during the infection of host cells, resulting in the disruption of cell monolayers in vitro [11,12]. Subsequently, this toxic factor was shown to be the AdV penton base protein [13], an outer capsid protein that contains an RGD sequence [14]. Mutations that alter the RGD sequence in the AdV2 penton base eliminated the cell rounding/detachment of this protein [15]. These observations suggested that the penton base was interacting with cell integrins and causing cell detachment rather than acting as a toxic component for cells.

### 1.1. Vitronectin-Binding Integrins αvβ3 and αvβ5 Promote AdV Uptake into Cells

In 1993, an investigation carried out in collaboration with David Cheresh, an expert in integrin cell biology [2], provided strong evidence that the vitronectin-binding integrins αvβ3 and αvβ5 were receptors for the AdV2 penton base [16]. Unlike conventional receptors, αv integrins were responsible for virus uptake into cells rather than initial virus attachment. Interestingly, the primary AdV2 attachment receptor, designated CAR (Coxsackie and Adenovirus Receptor) was not identified until 1997 by Jeff Bergelson and his colleagues at the University of Pennsylvania [17].

Evidence for integrin involvement in virus uptake was obtained by using complementary cell biological and molecular genetic approaches. Human M21-L4 melanoma cells expressing αvβ3 and αvβ5 integrins supported AdV infection as well as virus uptake, whereas M21-L12 cells lacking these integrins did not [16]. Function-blocking monoclonal antibodies to αvβ3 and αvβ5, as well as synthetic RGD integrin-binding peptides but not RGE (non-integrin-binding) peptides, interfered with AdV internalization into cells. And RGD integrin antagonists had no effect on virus attachment, consistent with the role of integrins in virus uptake rather than cell binding [16]. Other vitronectin-binding integrins, αvβ6 and αvβ8, function as receptors for mouse adenovirus-1 and adenovirus-3 but not for human adenoviruses [18].

The expression of a soluble form of recombinant αvβ5 integrin, comprising the entire extracellular domain, enabled a demonstration of its direct binding to AdV [19]. Soluble αvβ5 integrin bound directly to the species C AdV2 and the AdV2 penton base, as well as to its natural ligand, vitronectin, but not to a distinct ECM, fibronectin in solid-phase binding assays. Several other AdV types belonging to species B (AdV3), E (AdV4), and D (AdV37) also bound to this integrin. Moreover, soluble recombinant αvβ5 competed for AdV2-mediated gene delivery to various human cell types in vitro, indicating its role in virus infection.

### 1.2. Cryo-EM Structure Analyses of AdV in Complex with Integrins

Seeing is said to be believing. This adage was put to the test by examining the structure of AdV in complex with various ligands including integrins. The foundation of these studies was established by the cryoelectron microscopy (cryoEM) structure analysis of AdV alone at 35 Å resolution by Phoebe Stewart and her colleagues [20], in which they used the crystal structure of the major outer AdV capsid protein, hexon [21], to determine the phases. Subsequently, improved cryoEM image reconstructions of AdV were achieved as part of a long-standing collaboration with Phoebe Stewart (Case Western Reserve University) and Vijay Reddy (University of Minnesota, Hormel Inst).

**Figure 3:**
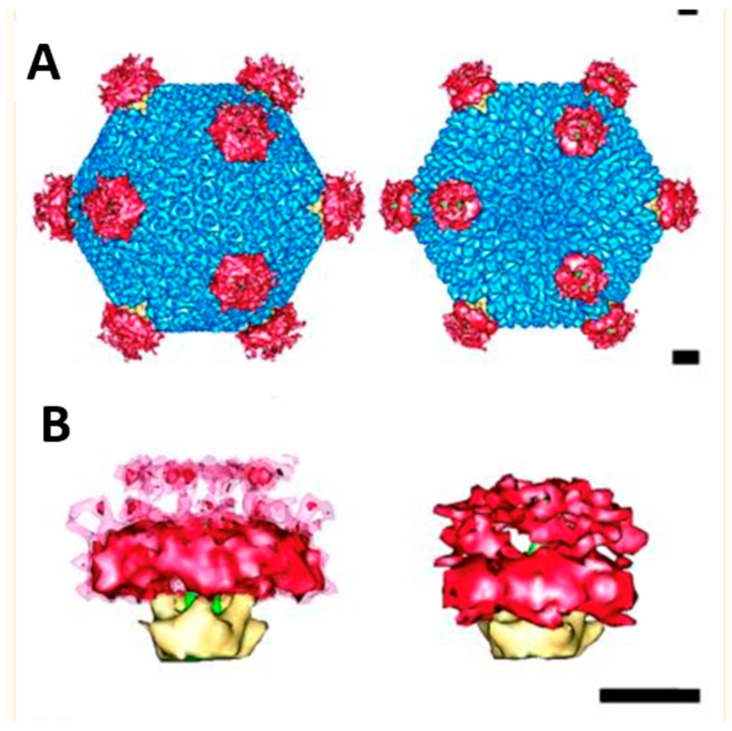
**Multimeric associations of AdV2 and Ad12 with integrin αvβ5.** CryoEM image reconstruction of Ad2 (**left**) and Ad12-integrin αvβ5 (**right**). In (**A**), the virus–integrin complexes are viewed along their icosahedral 3-fold axis. The hexons of the Ad particles are depicted in blue, the integrin is in red, and the penton base is in gold. In (**B**), the penton–integrin complexes are shown in-side view with the N-terminus of the fiber shaft (left) shown in green. The scale bars are 100 Å. Reprinted by permission from Chiu et al. 1999, Wiley publications [23].

The locations of the integrin-binding RGD sites on the Ad2 were determined by image reconstruction of the AdV in a complex with a monoclonal antibody directed against the penton base I**RGD**TRATR sequence [22]. Each penton base was shown to have five extended loops of ~22 Å with exposed RGD motifs. Fab fragments derived from the RGD-binding monoclonal antibody but not the intact antibody neutralized the virus in vitro. This suggested that the close spacing of the integrin-binding loops of the penton base, as well as the presence of the proximal N-terminal fiber domain, did not permit complete antibody saturation of all five RGD sites. Support for this hypothesis was obtained in subsequent BIAcore binding analyses in which only ~1.7 intact IgG antibodies bound to each penton base on the virus particle, whereas ~4.2 Fab fragments of the same antibody could bind [22]. Importantly, the cryoEM structure determination of AdV12, a virus that has a relatively short RGD loop comprising 17 amino acids, in complex with integrin αvβ5 revealed that integrins bind in a multimeric array (~4–5 molecules per penton base) [23] (Figure 3). The ability of adenoviruses to bind integrins in this multivalent format has also been observed with an AdV9 penton base and integrin αvβ3 [24]. Integrin clustering on the virus surface may be due to the ability of the integrins to adopt slightly different conformations, thereby maximizing their close spatial orientations on the virus. Thus, the AdV structure favors the binding of αv integrins while limiting the binding of potentially neutralizing antibodies.

## 2. Consequences of Integrin Binding to Adenovirus Particles

The clustering of four–five integrins by multiple penton base proteins displayed at each of the 12 vertices of the icosahedral AdV particle mediates several important functions for virus entry into host cells. Integrin clustering likely promotes cell-signaling processes that activate AdV endocytosis. In support of this concept, the penton base with its five RGD loops causes the activation of SyK kinase in lymphoid cells, whereas monomeric RGD peptides do not [25]. Importantly, the endocytosis of AdV via cell integrins was shown to accompany the activation of phosphoinositide-3-OH kinase (PI3K), and this signaling event was required for virion uptake into cells [26]. This lipid kinase interfaces with downstream activators of the cell cytoskeleton, such as the Rho family of GTPases, and the Rho family cell-signaling molecules, Rac1 and CDC42, promote AdV uptake [27].

Integrin clustering by the penton base provides another beneficial function for virus entry. The binding of multiple integrins appears to loosen the vertex of the viral capsid, thus favoring the release of the membrane lytic protein VI molecule from inside the virion [28,29]. Protein VI plays a crucial role in disruption of the cell endosome, allowing for the escape of internalized virions into the cell cytosol [30,31].

The continuing improvement in cryoEM techniques and associated CCD cameras have led to an even greater understanding of the AdV structure and the basis for virus interactions with integrins and other host molecules [32,33,34]. A more detailed cryoEM analysis of integrin αvβ5 bound to each RGD loop in the penton base of AdV12 suggested that the binding of this receptor induces a conformational change in the penton, which facilitates the disassembly of the entire vertex region of the virus [35].

These cryoEM structural studies are consistent with recent cell biological studies of AdV cell entry [36]. Early AdV capsid disassembly begins at the cell surface and elicits a cell-signaling response involving calcium flux into the cell, leading to the generation of sphingomyelinase. This enzyme produces ceramide, which accumulates in endocytic vesicles containing internalized adenovirus particles, thereby promoting endosome rupture by protein VI.

### The Role of Different αv Integrins and Other Integrin Types in Human AdV Cell Entry

As noted above, the vitronectin-binding integrins αvβ3 and αvβ5 are the major receptors involved in AdV uptake into cells. And integrin αvβ5 has been reported to have a unique role in endocytosis and the degradation of its natural ligand, vitronectin [37]. Moreover, integrin αvβ5 was found to have a selective role in promoting AdV-mediated membrane penetration [38]. In addition to αvβ3 and αvβ5, integrin αvβ1 can also facilitate AdV entry into certain transformed cell lines such as HEK293, on which integrins αvβ3 and αvβ5 are poorly expressed [39].

Besides the vitronectin-binding integrins αvβ1, αvβ3, and αvβ5, other types of integrins can also interact with adenoviruses and promote infection. One of these is integrin α_M_β2 [40]. This receptor is a member of the β2 integrin family and is highly expressed on hematopoietic cells, including monocyte/macrophages. Interestingly, α_M_β2 acts as an attachment receptor for adenovirus cell attachment. Thus, virus binding is inhibited by the presence of a soluble penton base or a function-blocking antibody to α_M_β2. The entry of the virus into monocyte/macrophages is enhanced by the presence of αv integrins.

Certain adenoviruses associated with severe ocular infections, such as AdV37, have been shown to use αvβ1 as well as α3β1 integrin to infect human corneal epithelial cells [41]. Since primary human corneal epithelial cells lack β3 and β5, each of these different β1 integrins could serve as either primary or secondary receptors or both on these cells.

Species F human adenoviruses, including types 40 and 41, are a frequent cause of gastrointestinal diseases. Strikingly, these viruses lack the RGD motif in their penton base, although they readily infect intestinal epithelial cells. Recent studies indicated that laminin-binding integrins containing the α6 subunit serve as a co-receptor for AdV40/41 uptake and infection [42].

Even though most adenovirus types use one or more integrin types, certain viruses do not employ integrins to invade host cells. A well-documented example of this is canine adenovirus type 2 (CAV2), which uses its fiber protein to interact with CAR-1 on both rodent and human neuronal cells [43]. The use of this primary attachment receptor is sufficient to promote the retrograde axonal transport of virions, a feature that might facilitate the use of this non-human virus as a vector for neurologic diseases [44].

It has also been reported that keratinocytes and airway epithelial cells derived from beta 5 integrin-deficient mice support similar levels of AdV5 infection as normal cells expressing this integrin [45]. It is possible that infection proceeds solely via CAR or perhaps in concert with other αv integrin co-receptors, such as αvβ1 or αvβ3. In related studies, primary cultured hepatocytes were shown to express only CAR but not αv integrins, and the adenovirus infection of these cells proceeded solely through fiber–CAR association [46]. The infection of liver hepatocytes in vivo is even more complex. In this case, plasma-derived clotting factors direct the virus to heparin sulfate moieties on the surface of these cells, thereby circumventing the use of CAR or integrins [47].

Although this is not the focus of this review, other diverse RNA and DNA viruses utilize integrins for infection. A few examples are the Epstein–Barr virus (EBV), which uses αvβ6 and αvβ8 to trigger fusion of its envelope with host cells [48]; foot-and-mouth disease virus (FMDV), which interacts with multiple αv integrins to achieve infection [49]; and hantaviruses, which bind to β3 integrins to infect human tissues and cause hemorrhagic fever with renal pathology [50].

## 3. Modifications of AdV to Alter Cell Tropism

As previously discussed, virus cell tropism is primarily mediated by penton base binding to αv integrins as well as fiber-mediated attachment to receptors such as CAR [51]. In general, this broad cell tropism is controlled by both types of receptors and cannot be completely abrogated by deleting either of these interactions with different receptors via mutagenesis. Instead, ablation of both interactions is required to reduce cell tropism [52]. This finding has potential clinical relevance for the use of AdV to treat human diseases including cancer. In general, AdV vectors injected intravenously or intraperitoneally have a propensity to be taken up in the liver, thus hindering the transduction and killing of tumor cells that may be located outside this organ. By contrast, removal of the CAR-binding sequences in the fiber and the integrin-binding RGD motif in the penton base greatly reduced AdV vector-targeting of the liver, as well as other tissues when administered intraperitoneally in a mouse model [53]. These findings raised the possibility of reengineering AdV vectors to avoid the natural tropism of the virus by ablating binding to its normal receptors, combined with the addition of targeting sequences that recognize molecules preferentially displayed on tumor cells, thereby increasing the specificity and efficiency of cell killing.

AdV vectors are now being reconfigured to circumvent normal receptor usage. This effort largely arose from the pioneering efforts of David Curiel and his colleagues. In one example, they modified the fiber protein of AdV5 by inserting an integrin-binding RGD motif as well as a poly-L-lysine motif into the HI-loop of the fiber knob [54,55]. The doubly modified vector lost its ability to bind to CAR in vitro while exhibiting a propensity to target nonhuman primate pancreatic islets compared to non-modified vectors.

This AdV fiber RGD insertion strategy has recently been extended in recent phase I human clinical trials for the treatment of glioblastoma [56]. In this study, more than a dozen enrolled subjects with recurrent glioblastoma received an oncolytic AdV5 displaying the fiber RGD sequence. The tumor selectivity of the Delta 24-RGD AdV5 vector was provided by the deletion of the 24 base pairs in the E1A genomic regions of the vector, while the RGD insertion facilitated integrin association with the tumor cells. Overall, this safety investigation revealed that the vector, administered directly into the brain, appeared to be safe, induced a local inflammatory reaction, and increased survival in a subset of the subjects. Further human trials will be necessary to substantiate these findings and to determine whether the inflammatory responses can be used to predict efficacy.

Similar strategies involving the insertion of RGD into outer capsid proteins of AdV vectors by other investigators appear to improve gene delivery to vascular smooth muscle cells, which are refractory to unmodified AdV5 [57]. Removal of the clotting factor X-binding region of the hexon in AdV5 and addition of an RGD peptide sequence improved gene transfer to these cells. However, these hexon-altered vectors that enhance gene transfer via an integrin-dependent manner were still susceptible to pre-existing neutralizing antibodies to the virus, presumably through antibody recognition of the hexon, penton base, and/or fiber. Thus, the in vivo efficacy of RGD-modified vectors delivered through the blood stream may be reduced due to their elimination by host immune factors such as antibodies.

In addition to smooth muscle vascular cells, endothelial cells can be targeted by AdV vectors that have been modified to adhere to integrins such as αvβ3 [58]. The asparagine, glycine, arginine (NGR) peptide sequence incorporated into the fiber of AdV5 or the hexon protein enhanced gene delivery to vascular endothelial cells in vitro. The NGR sequence recognizes integrins when presented as a linear peptide or amino peptidases (CD13) when it is displayed as a circular molecule. In a similar way, NGR peptides are used for ligand-directed delivery of different therapeutic molecules to neoplastic tissue, especially those associated with angiogenesis [59].

Other specific human cell types that are generally refractory to transduction by AdV5-based vectors may also be amenable to gene transfer using RGD-modified vectors. For example, both human and murine T cells, which lack the primary receptor CAR but have low levels of both αvβ3 and αvβ5, are generally resistant to adenovirus infection. However, these cells can be transduced with a vector containing an RGD-modified fiber, which is also viral replication-competent [60]. These findings offer the possibility of a pathway toward the gene transfer of oncolytic adenoviruses to treat cancers arising in hemopoietic cell types. In an expansion of this approach, AdV-transduced T cells were used as a reservoir of oncolytic virus to infiltrate glioblastomas in the brain [61]. In this study, a Jurkat T-cell line was infected with an oncolytic AdV vector bearing an RGD-modified fiber. These T cells were then injected into the brains of mice bearing tumors derived from the transplantation of a glioblastoma stem cell line, MGG8. This led to a restriction of tumor growth and better survival. Whether these preclinical advances can be extended to humans and if they have an acceptable safety profile remains to be determined. In addition, the production of clinical-grade RGD-modified AdV vectors has been somewhat stymied by their propensity to aggregate during virion purification in cesium chloride gradients. This is likely due to the cross-linking of free sulfhydryl residues in the inserted RGD peptide, a problem that can be circumvented by using iodixanol gradient centrifugation [62].

### 3.1. Integrin αvβ6 as a Novel Target for AdV Cancer Therapy

While integrins αvβ3, αvβ1, and αvβ5 are widely expressed on normal, healthy epithelial cells, integrin αvβ6 is absent or present at very low levels on these cell types [63]. By contrast, keratinocytes that proliferate during the process of wound healing express integrin αvβ6 concomitantly with its natural ligands’ fibronectin and tenascin [64]. Integrin αvβ6 is also highly upregulated on different cancer cells [65], a situation that has provided an opportunity to target cancer tissue with reengineered AdV vectors. However, most human AdV vectors do not efficiently recognize this integrin αvβ6. This is likely because the RGD present in the HAdV penton base is displayed in a different context than that recognized by αvβ6. This was revealed by studies of several murine Ads (MAV-1 and MAV-3), which have a different RGD flanking sequence, RGDLXXL(I), than that present in human AdV. This distinct RGD sequence allows murine adenovirus type 1 to usurp αvβ6 integrins for infection of various cells in the central nervous system of C57/Bl6 mice [66]. Furthermore, incorporation of the RGDLXXL sequence into a human AdV allowed for efficient gene transfer to cells expressing integrin αvβ6 [67].

Re-designed AdV vectors capable of targeting αvβ6 integrin have recently been investigated in preclinical studies for cancer therapy. In one study, an oncolytic Adv5 vector containing deletions in the hexon, penton base, and fiber that ablates virus association with CAR, integrins αvβ3 and αvβ5, as well as clotting factors, was equipped with a 20mer peptide (A20, NAVPNLRGDLQLAQKVART) inserted into the HI loop of the fiber knob [68,69]. This RGD peptide sequence originally came from the GH loop of VP1 in the foot-and-mouth disease virus (FMDV) that avidly associates with the αvβ6 integrin. Intraperitoneal delivery of the αvβ6-targeted oncolytic Ad5Null-A20 vector allowed for the infection of metastatic tumor tissue in a mouse model of ovarian cancer, and this resulted in enhanced survival relative to mice receiving a control vector [70].

Targeting of integrin αvβ6 may be useful for eliminating other types of tumors, including those arising in the pancreas [71,72]. In this regard, an oncolytic AdV5-based vector ablated for CAR binding and containing the FMDV-derived RGD peptide inserted into the fiber allowed for the efficient killing of αvβ6 integrin-expressing pancreatic cancer cells [71]. Moreover, radiolabeled versions of this oncolytic virus were used to determine the biodistribution of pancreatic tumor cells in mouse models, an approach that could presage tumor imaging in human subjects [72].

### 3.2. Equipping Other Viruses and Non-Viral Nanoparticles with RGD Sequences

Although somewhat beyond the focus of this adenovirus-centric review, it is relevant to mention that similar strategies for cancer therapy employ other viruses and nanoparticles to target integrins expressed on malignant tissues.

An example of this is a baculovirus vector-based *Autographa californica* multiple nucleopolyhedrovirus [73]. To target integrins on neoplastic cells, RGD sequences found in the C-terminus of Coxsackie virus A9 and parechovirus 1 were fused to one of the major envelope glycoproteins of the baculovirus vector. This RGD-modified virus was capable of binding to A549 epithelial cells that express αv integrins relative to poor or no binding by the unmodified baculovirus. In addition, the presence of the RGD sequence in the modified vector provided a significant increase in cell transduction.

**Figure 4:**
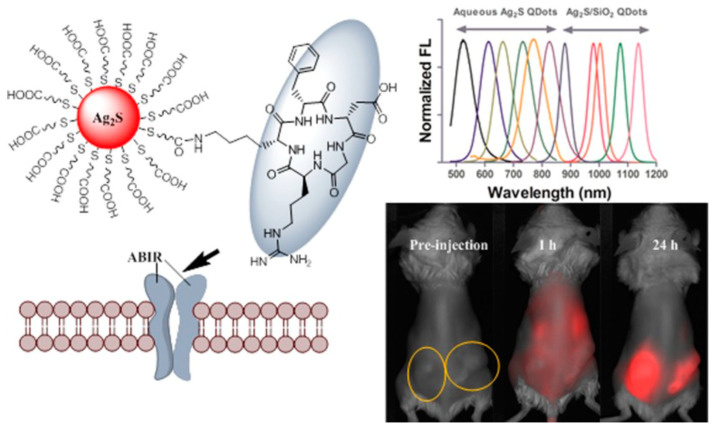
**Design of fluorescent silver sulfide nanoparticles displaying RGD integrin-targeting sequences**. Nanoparticles known as quantum dots (orange) of ~1–10 nm with distinct tunable light emission spectra from 500–1200 nm can be derivatized with multiple RGD peptides (outlined in grey oval) to target various integrins (ABIR; alpha beta integrin receptor) depicted in the membrane cartoon (lower left). These nanoparticles, when administered in mouse models, show preferential tumor accumulation over time (lower right panel). Reprinted with permission from Tang et al. copyright 2015, ACS publications [75].

An even greater expansion of the integrin-targeting strategies involves the use of synthetic nanoparticles displaying RGD sequences for bioimaging and the therapeutic treatment of cancers [74]. To image small clusters of tumor cells in vivo, nanoparticles comprising gold, silver sulfide, iron oxide, palladium, or technetium were coated with various forms of the RGD peptide to target αv integrins on the tumor cells, as well as on neovascular (angiogenic) tissues [74]. These novel nanoparticles provided a more accurate localization of minute tumor masses [75] (Figure 4).

RGD-coated nanoparticles not only have the potential to enhance tumor imaging, but they also might be able to eradicate cancer. In one example, RGD-coated nanoparticles having the ability to bind to integrin αvβ3 were loaded with an anti-tumor agent, paclitaxel, to kill targeted tumor cells in vivo [76]. The combination of more selective tumor targeting by virtue of displaying the RGD motif as well as subsequent killing by paclitaxel appeared to enhance survival in tumor-bearing mice.

Besides metal-based nanoparticles, lipid-based (liposomes), derivatized with RGD peptides, are also being investigated [77]. The advantage of using liposomes as a clinic modality is that these particles can carry both hydrophilic and lipophilic drugs with inherent controlled release and with increased half-life in the circulation, as well as a satisfactory safety profile. And like metal-based nanoparticles, they can be readily equipped with integrin-binding RGD peptides. One of the first liposome-delivered drugs was a product known as Doxil, which carried doxorubicin, an anti-tumor molecule that was originally approved by the FDA in 1995 [78]. Subsequently, other liposome formulations such as those carrying paclitaxel have entered phase II clinical trials [79]. As with metal-based nanoparticles bearing RGD sequences, liposomes displaying various RGD peptide sequences have also been deployed to enhance the delivery of doxorubicin or paclitaxel, resulting in increased survival in mouse models of C26 carcinoma [80] or glioma, respectively [81].

## 4. Conclusions and Future Endeavors

The identification of a “toxic factor” produced during adenovirus infection led to the discovery that many adenoviruses use integrins as receptors for virus uptake into cells. Recognition of the vitronectin-binding integrins is mediated by RGD peptide sequences present in the AdV penton base of most but not all species of adenovirus. These vitronectin-binding integrins play a major role in cell entry by triggering intracellular signaling pathways upon penton base ligation. They also facilitate capsid disassembly and the release of protein VI, beginning at the cell surface and finishing in the early endosome. Biochemical analyses combined with cryoEM structural studies revealed multivalent associations of αvβ5 integrin with the adenovirus penton base. These fundamental research findings have provided an opportunity to reengineer adenoviral vectors for bioimaging and potentially for the eradication of solid tumors.

In one clinical application, AdV peptide sequences that mediate the virus attachment receptor (CAR), as well as sequences involved in αvβ3 and αvβ5 integrin association, were deleted. These changes were then combined with the insertion of a specific RGD peptide into the fiber protein that selectively recognizes integrin αvβ6, a receptor that is highly expressed on neoplastic tissue. This retargeted viral vector enabled improved efficacy for the treatment of glioblastoma in preclinical and early phase I trials. Similar efforts are being brought forth with the use of other viruses, as well as metallic-based nanoparticles and other synthetic compositions. Despite these technical advances, it remains to be determined how effective these strategies will be in treating different metastatic cancers and the extent to which the adaptive and innate host immune will limit vector efficacy. Nonetheless, in the coming years, we are likely to see more examples of AdV engineering, including those that limit vector association with neutralizing antibodies, clotting factors, and other host molecules to improve their tissue specificity and safety. A recent example of this is the use of oncolytic Ad vectors displaying the RGD-binding motif for integrin αvβ6 that are also coated with cationic nanoparticles [82]. These cationic moieties serve to limit vector association with clotting factors.

Additional sites in the AdV vector may be suitable for the insertion of RGD-targeting motifs. For example, an RGD sequence was inserted into the hypervariable region 5 (HVR5) in the hexon to improve vector targeting in smooth muscle tissue [83]. Other non-viral forms of adenovirus that have higher safety profiles than those of intact AdV particles might also serve as a platform for cell targeting. One of those is the AdV dodecahedron, a multivalent complex of the virus penton base and/or fiber protein that maintains the ability to recognize cell integrins [84]. A less well-known platform for cell targeting may be vault nanoparticles. Vaults are natural constituents of host cells that can be generated as recombinant virus-like particles [85,86]. Recombinant vault nanoparticles have already been equipped with the adenovirus protein VI lytic peptide [87,88], as well as RGD peptides [89], to enhance cell transduction. These engineered synthetic and recombinant cell-based platforms may eventually supplant AdV vectors as sustainable drug carriers.

## Data Availability

No new data were included in this manuscript.
